# The Main Role of Diaphragm Muscle as a Mechanism of Hypopressive Abdominal Gymnastics to Improve Non-Specific Chronic Low Back Pain: A Randomized Controlled Trial

**DOI:** 10.3390/jcm10214983

**Published:** 2021-10-27

**Authors:** Davinia Vicente-Campos, Sandra Sanchez-Jorge, Pablo Terrón-Manrique, Marion Guisard, Marion Collin, Borja Castaño, David Rodríguez-Sanz, Ricardo Becerro-de-Bengoa-Vallejo, José López Chicharro, César Calvo-Lobo

**Affiliations:** 1 Faculty of Health Sciences, Universidad Francisco de Vitoria, Pozuelo de Alarcón, 28223 Madrid, Spain; davinia.vicente@ufv.es (D.V.-C.); s.sjorge.prof@ufv.es (S.S.-J.); p.terron.prof@ufv.es (P.T.-M.); marion.guisard@gmail.com (M.G.); marionccollin@gmail.com (M.C.); borjakast@gmail.com (B.C.); 2 Faculty of Nursing, Physiotherapy and Podiatry, Universidad Complutense de Madrid, 28040 Madrid, Spain; davidrodriguezsanz@ucm.es (D.R.-S.); ribebeva@enf.ucm.es (R.B.-d.-B.-V.); cescalvo@ucm.es (C.C.-L.); 3 Grupo FEBIO, Universidad Complutense de Madrid, 28040 Madrid, Spain

**Keywords:** back pain, hypopressive exercises, diaphragm thickness, diaphragm strength, RUSI

## Abstract

Background: Chronic low back pain (LBP) has been stated as one of the main health concerns in the XXI century due to its high incidence. Objective: The objective of this study was to determine the effects of an 8-week program of hypopressive abdominal gymnastics (HAG) on inspiratory muscle strength, diaphragm thickness, disability and pain in patients suffering from non-specific chronic LBP. Methods: A total of 40 patients with chronic LBP were randomly divided into two groups. The experimental group carried out an 8-week supervised program of HAG (two sessions/week), whereas the control group did not receive any treatment. Outcomes were measured before and after the intervention, comprising diaphragm thickness during relaxed respiratory activity, maximal inspiratory pressure (PI_max_), pain intensity (NRS), pressure pain threshold and responses to four questionnaires: Physical Activity Questionnaire (PAQ), Roland–Morris Disability Questionnaire (RMQ), Central Sensitization Inventory (CSI) and Tampa Scale of Kinesiophobia-11 Items (TSK-11). Results: Statistically significant differences (*p* < 0.05) were observed for greater thickness of the left and right hemi-diaphragms at inspiration, as well as higher PI_max_ and decreased NRS, CSI and RMQ scores in the intervention group. After treatment, the increases in the thickness of the left and right hemi-diaphragms at inspiration and PI_max_, as well as the decrease in the NRS and RMQ scores, were only predicted by the proposed intervention (*R*^2^ = 0.118–0.552). Conclusions: An 8-week HAG intervention seemed to show beneficial effects and predicted an increase in diaphragm thickness and strength during inspiration, as well as a reduction in pain intensity, central sensitization and disability, in patients suffering from chronic non-specific LBP with respect to non-intervention.

## 1. Introduction

Currently, chronic low back pain (LBP) has been stated as one of the main health problems in the XXI century due to its high incidence, being, at the same time, one of the most disabling conditions in healthy adults [1,2,3]. According to its etiology, chronic LBP may be categorized into specific and non-specific types. Specific LBP may be identified secondary to a root cause, while non-specific LBP may be diagnosed if a patient’s pain origin is lacking. Approximately 90% of LBP conditions may be classified as the non-specific type. In addition, from 2% to 7% of these patients suffer from chronic LBP which interferes with their functional abilities, thus affecting their quality of life [4].

The term “core” is defined as a three-dimensional space within muscular boundaries, such as the diaphragm providing the upper limit, rectus abdominis and oblique musculature as the anterior-lateral limits, gluteal and paraspinal muscles forming the posterior limit and pelvic floor muscles as the lower limit. The inherent nature of these muscular edges may produce, by their co-contraction, a stabilization effect in the spine, being relevant to correctly perform trunk and limb movements [5]. The key role of the diaphragm in stabilizing the trunk has been researched for more than a half century, but the concrete mechanisms are still poorly understood [6,7]. The diaphragm, together with the abdominal muscles, may generate hydraulic effects in the abdominal cavity that may assist in spine stabilization [8,9,10], maintaining the lower spine through intra-abdominal pressure increases [10]. Indeed, both the diaphragm and pelvic floor were considered as synergistic muscles with respect to the transversus abdominis for the increase in this intra-abdominal pressure and the maintenance of different postures [11]. In 2012, Kolar et al. observed that a comparison between subjects with and without LBP presented abnormal positions of the diaphragm muscle and a lower curvature of the diaphragm, which could contribute to the etiology of this condition [12].

Physical therapy has presented a wide variety of interventions whose final purpose is the treatment and functional recovery of LBP-affected patients. Training of the muscles which generates trunk stability may often help to improve LBP [13,14,15,16]. One of these training methods is hypopressive abdominal gymnastics (HAG), which is becoming increasingly popular. In 1980, Caufriez developed training series composed of 33 hypopressive exercises associated with a different posture, such as seated, kneeling, quadruped and supine. Each exercise was combined with a hypopressive maneuver comprising an apnea after a prolonged exhalation due to the breath being held at the end of exhalation. These exercises were performed while “sucking in his/her abdomen” and opening the ribs by means of an intense and voluntary contraction of the accessory inspiratory musculature, such as the serratus anterior, external intercostalis, scalenes and sternocleidomastoid, keeping the glottis closed, known as “diaphragmatic suction”. According to HAG, these exercises may produce a direct activation of transversus abdominis muscle, which could strengthen the abdominal wall and stabilize the spine [17,18,19]. Thus, the mechanisms that explain how the proposed exercises affect the transversus abdominis are well known. Nevertheless, the role of the diaphragm during HAG remains unclear, and the previously described “diaphragmatic suction” should imply this muscle as the target muscle during this technique.

The main indications of HAG are the treatment of abdominal diastasis in the postpartum period, urinary incontinence and pelvic prolapse, as well as chronic LBP [17]. To date, the majority of studies have focused on assessing the effects of HAG on pelvic floor dysfunctions [20,21,22]. Nevertheless, very few studies have assessed the effects on other core muscle groups which form part of the core and which may help to stabilize the spine in a correct way [17,19]. To the best of our knowledge, there is a lack of studies assessing the effects of HAG on the diaphragm as the key muscle that forms the upper core wall.

Indeed, the rehabilitative ultrasound imaging (RUSI) technique was used for both static and dynamic assessment of the abdominal wall, multifidus and pelvic floor muscles [23] in both athletes and patients with LBP. Nevertheless, novel research assessing diaphragm morphology and muscle activity in patients with chronic LBP is lacking. B-mode was tested and validated to assess diaphragm morphology during respiration by the trans-costal RUSI technique [24,25,26]. In 2013, Vostatek et al. showed thinner diaphragms in patients with lumbopelvic pain [27]. In 2019, similarly, Calvo-Lobo et al. [28] observed a smaller diaphragm thickness in athletes suffering from non-specific lumbopelvic pain with respect to athletes without pain, suggesting that training of this muscle should be a key focus of treatment in relation to sports prevention, performance and rehabilitation. Therefore, the aim of the present study was to determine the effects of an 8-week program of HAG on inspiratory muscle strength, diaphragm thickness, disability and pain in patients suffering from non-specific chronic LBP.

## 2. Methods

### 2.1. Study Design

The study design comprised a single-blinded, randomized controlled trial (blinded examiner) including patients diagnosed with non-specific chronic LBP. Information on this trial was reported in adherence with the CONSORT checklist [29]. The ethics committee of Francisco de Vitoria University approved this study (approval code 1/2021), which was registered on clinicaltrials.gov (NCT04750187). All participating patients provided written informed consent.

### 2.2. Sample Size Calculation

The sample size calculation was obtained by G*Power 3.1.9.2 (Universität Düsseldorf, Düsseldorf, Germany) applying a *t*-test family calculation for the means difference of 2 independent groups using a power analysis for a priori sample size calculation with an alpha of 0.05, a power of 0.80 and a large effect size of *d* > 0.80 [30]. Thus, a sample size of 40 patients, 20 patients for each group, was required.

### 2.3. Participants

A consecutive convenience sampling method was applied in order to recruit 40 non-specific chronic LBP patients from the Francisco de Vitoria University, with a prior diagnosis of chronic LBP of non-specific origin carried out by a medical doctor. Patients were divided into 2 groups: (1) experimental group who underwent 8 weeks of HAG, and (2) control group who did not receive any treatment. The inclusion criteria were a prior medical diagnosis of non-specific chronic LBP, considering the presence of non-specific origin pain mainly located between the subcostal line and the bi-iliac line for at least 3 episodes in the last 6 months and reporting at least 10% on the Oswestry disability scale [31]. Exclusion criteria included presence of lumbopelvic musculoskeletal conditions (at least for the previous year) or congenital abnormalities, neuromuscular conditions (different from non-specific chronic LBP) or rheumatisms, body mass index greater than 31 kg/m^2^, previous diagnosis considering neurological or respiratory pathologies, surgery and trunk alterations, skin conditions, inability to follow some instructions for the research course and pregnancy [32].

### 2.4. Procedures

At the beginning of the study, all participants underwent a physical examination by an experienced physiotherapist to confirm the possibility of participating in the study. Demographic data were collected by a questionnaire. Height (cm) and weight (kg) were measured for each participant, and body mass index (BMI) was calculated.

#### Hypopressive Abdominal Gymnastics (HAG)

The participants included in the experimental group carried out 2 sessions of HAG per week, for 8 weeks. The sessions lasted between 30 and 40 min and were always supervised by a physiotherapist with more than 4 years of clinical experience in HAG.

HAG was carried out respecting the principles described by Caufriez [33], and Rebullido and Pinsach [34], who detailed the following steps: (a) neutral pelvis as well as spine elongation; (b) dorsiflexion of the ankles; (c) flexion of the knees; (d) shoulder girdle muscle activation; (e) 3 breathing cycles completed with lateral-costal breathing as well as slow deep exhalations (inspiration and maximum exhalation); (f) breathing maintenance after expansion of the rib cage (“diaphragmatic aspiration”).

Each session consisted of 6 hypopressive abdominal exercises separated by 2 min of recovery between them, and each exercise was repeated 3 times. All the study participants received a period of familiarization and learned how to perform the “diaphragmatic aspiration” maneuver, prior to the beginning of the training period, which consisted of exhaling all the air until reaching the reserve volume, then holding their breath (apnea) and opening the ribs, drawing the abdominal wall inward as well as cranially without letting air in [18]. All participants were asked for non-contraction of their abdomen voluntarily throughout the sessions. A description of each exercise completed with figures is included in Supplemental File S1.

The participants included in the control group were asked to continue their life as usual, without changing their usual physical activity.

### 2.5. Outcome Measures

#### 2.5.1. Diaphragm Thickness

A high-quality ultrasonography tool named Toshiba Xario 100 (Toshiba, Madrid, Spain) was used to measure the diaphragm thickness. All measurements were performed by a specialized physical therapist with more than 4 years of clinical experience in ultrasound imaging, who was blinded to groups (experimental or control) due to all participants being assigned codes for measurements, and the operator was blinded to subjects’ allocation to the 2 groups. A linear probe (named PLT-805AT Toshiba, Toshiba, Madrid, Spain) with a range of frequencies from 8 to 12.0 MHz as well as a 45 mm footprint was used to carry out the trans-costal measurements at rest and placed in supine decubitus using B-mode ultrasound images (with a pre-fixed preset including 3 cm of depth, 12.0 MHz of frequency, 64 points of dynamic range and 64 points of gain, and one focus placed at 2 cm of depth) [28]. After, static grayscale images for measurements were stored as Digital Imaging and Communications in Medicine (DICOM) and transferred to a computer as well as being calibrated to centimeters (cm). Diaphragm thickness was evaluated for relaxed respiratory activity (at final inspiration, at final expiration and their difference) using trans-costal ultrasonography.

**Figure 1:**
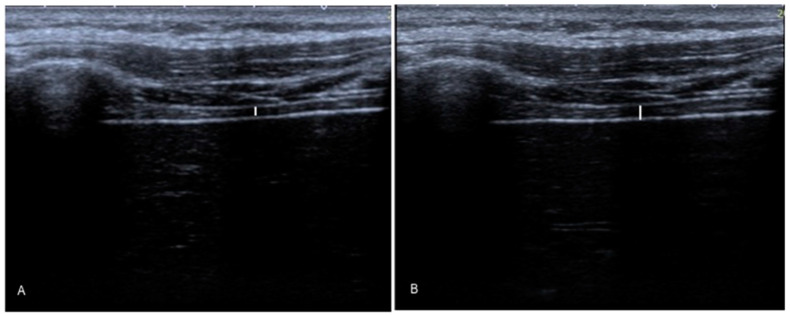
Ultrasound imaging of the hemi-diaphragm by linear probe at the final relaxed expiration (T^exp^; **A**) and final relaxed inspiration (T^ins^; **B**).

Images of bilateral trans-costal ultrasonography images were taken using a linear probe located perpendicularly with respect to the lowest intercostal space (according to the mid-axillary line considered from the 12th rib cranial edge to the 11^th^ rib caudal edge), which allowed the correct diaphragm visualization without lung encroachment for tidal breathing. The diaphragm muscle is bilaterally placed deep in the intercostal musculature layer and ribs. Three images were captured for each hemi-diaphragm for final relaxed expiration (T^exp^; Figure 1A), and three images were captured for each hemi-diaphragm for final relaxed inspiration (T^ins^; Figure 1B). Bilateral diaphragm musculature thickness was measured by placing electronic calipers inside of the 2 hyperechoic lines of the peri-muscular connective tissues that outlines the diaphragm placed at the intercostal space center. The three repeated measurements’ mean was analyzed for each hemi-diaphragm to detail the thickness during final relaxed inspiration (T^ins^) as well as final relaxed expiration (T^exp^), and their differences (T^ins^-T^exp^). Excellent inter-rater reliability properties showing high intraclass correlation coefficients (ICCs) were reported for T^ins^ (ICC of 0.94; 95% CI from 0.91 to 0.99) and T^exp^ (ICC of 0.98; 95% CI from 0.94 to 0.99) measurements following previous reliability analyses performed by Harper et al. [24].

#### 2.5.2. Inspiratory Muscle Strength

Inspiratory muscle strength was assessed by assessing PI_max_ applying a POWERbreathe^®^ KH1 device (Powerbreathe International Ltd., Warwickshire, United Kingdom) from residual volume, following the rules from the American Thoracic Society and European Respiratory Society [35,36]. Each measurement was obtained in the reference unit of centimeters of water columns (cmH_2_O). The procedures were repeated at least three times or until two reproducible efforts (with 5% of each other). Intervals of 1 min were allowed between measurements in order to avoid short-term fatigue appearance of the respiratory musculature. The highest of the two reproducible values was used for data analyses [37].

#### 2.5.3. Pain Intensity

Pain intensity was measured by the Numerical Rating Scale (NRS) as a numerical pain scale. This tool evaluates pain intensity from 0 which corresponds to “no pain” to 10 which corresponds to “pain of maximum intensity”. This scale was applied for the evaluation of pain intensity in a quantitative way [38].

#### 2.5.4. Pain Threshold

The pressure pain threshold was measured using a digital handheld algometer with a stimulation area of 1cm^2^ (FDK/FDN, Wagner Instruments, 1217 Greenwich, CT 06836). The stimulation surface was placed on the spinous process of the L4 lumbar vertebra, whose evaluation has shown a good intraclass correlation coefficient (ICC > 0.75) [39]. Three consecutive measurements were performed with 1 min of recovery between them, and the average of them was used for the data analyses. The participants were placed in a prone position, and the exact measurement point was marked to ensure that the repeated measurements were carried out at the same point; then, the measurement began with a pressure-generating speed of 50 kPa/s. During the measurement, the participants said “stop” when they began to feel pain. Before the measurement, the participants received standardized instructions as follows: “The pressure is going to gradually increase. Allow the pressure to build until it reaches the point where you feel pain or discomfort, and then say “stop”. This means that it indicates the moment in which you begin to feel pain, not the maximum pain that you are able to endure” [40].

#### 2.5.5. Questionnaires

Participants responded to 4 previously validated questionnaires:

− Physical Activity Questionnaire (IPAQ). This tool is formed of 4 questions about specific types of physical activities, for example, walking as well as vigorous and moderate activities, the frequency as well as the duration of each specific activity type and the time spent seated per day in each week. Data calculated using the IPAQ were converted into MET-min/week (named metabolic equivalents) by calculating the minutes per week for each category of the activities within their specific metabolic equivalents (walking corresponded to 3.3 METs; moderate physical activity corresponded to 4 METs; vigorous physical activity corresponded to 8 METs). Physical activity levels for each individual were ranked following IPAQ’s recommendations, which describe the physical activity categories as follows: Category I (considered as low physical activity) that corresponds to individuals who do not fulfil the criteria of the other 2 categories, considered as inactive; Category II (considered as moderate physical activity) that corresponds to individuals who meet 1 of the following criteria: 3 and/or more days of vigorous physical activity for at least 20 min per day, or 5 and/or more days of any combination of walking and vigorous or moderate physical activity, which reaches a total physical activity of at least 600 MET-min/week; Category II (considered as high physical activity) that corresponds to subjects who met 1 of the following criteria: vigorous activity of at least 5 days, reaching a total physical activity of 1500 MET-min/week, or 7 and/or more days of any combination of walking and moderate and/or vigorous activity that reaches a total physical activity of at least 3000 MET-min/week.
− Roland–Morris Disability Questionnaire (RMDQ). This reliable and validated questionnaire presents 24 items which measure limitations under daily life activities secondary to LBP. The RMDQ Spanish version presents good comprehensibility, reliability and internal consistency and is considered as a useful and adequate instrument to determine disability originated by LBP [41].
− Central Sensitization Inventory (CSI). This questionnaire allows the identification of symptoms related to central sensitization. This tool consists of two parts. The first part of this questionnaire analyzes 25 symptoms scored from 4 (always) to 0 (never), the total score being from 0 to 100 points. A score greater than 40 may be considered as the cut-off for detecting a central sensitization syndrome. The second part consists of questions about possible conditions that patients have been previously diagnosed with related to central sensitization [20].
− Tampa Scale of Kinesiophobia-11 Items (TSK-11). The Spanish adaptation of the TSK-11 scale was used. The literature has demonstrated the validity and reliability of this test in assessing the level of kinesiophobia, especially for patients with chronic non-specific LBP. Unlike the original TSK, the TSK-11 is an abbreviated version with only eleven items, with four possible responses: strongly agree, agree, disagree and strongly disagree. The scoring range varies from 11 to 44 points, with some articles considering a significant difference as a difference of 4 points after treatment [20].

### 2.6. Statistical Analysis

SPSS 24v (IBM; Armonk–NY; IBM–Corp) was utilized to carry out all statistical analyses with an alpha error of 0.05 considering a statistically significant *p*-value of <0.05, with a 95% confidence interval (CI).

The Shapiro–Wilk test was applied to determine normality. All data were recorded as mean ± standard deviation (SD), mean difference and the lower and upper limits of the 95% CI, and *t* or *U* statistics for parametric or non-parametric data, respectively.

Between-group comparisons were analyzed by the independent sample Student *t*-test or Mann–Whitney *U* test for parametric or non-parametric data, respectively. Regarding between-group outcome measurement differences, effect sizes were determined by Cohen’s *d* and interpreted as very small (*d* < 0.20), small (*d* from 0.20 to 0.49), medium (*d* from 0.50 to 0.79) or large (*d* > 0.8) [42]. In addition, sex was recorded as a percentage (%) and frequency (*n*) and compared by the Fisher exact test.

Finally, multivariate linear regression analyses were carried out by the stepwise selection method in order to predict the outcome measurements which showed between-group differences. *R*^2^ coefficients were used to determine the adjustment quality. Baseline descriptive data and outcome measurements (different from the predicted variable) were included as independent variables. The outcome measurements, which showed between-group differences, were included as dependent variables for each prediction model.

## 3. Results

### 3.1. Baseline Data

**Figure 2:**
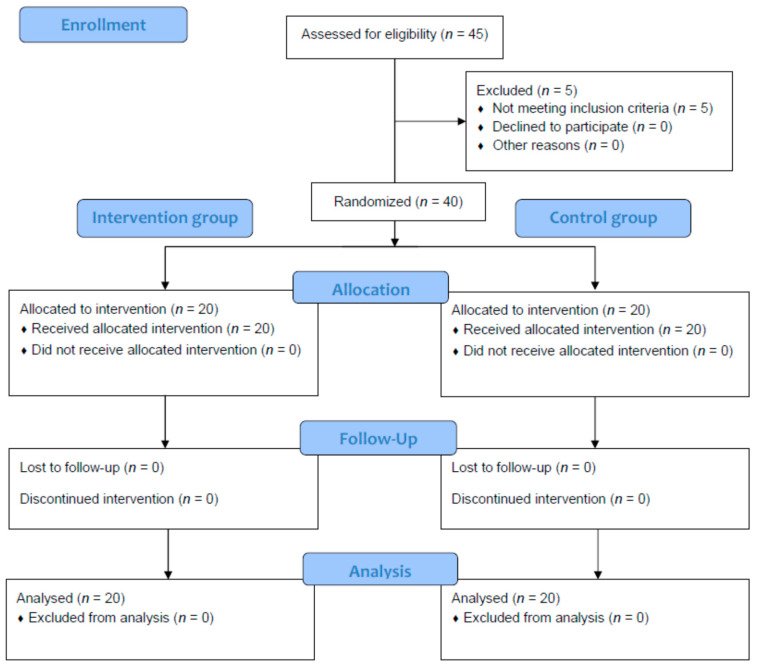
Flowchart of the trial.

The flowchart of the trial is presented in Figure 2. Baseline descriptive data and outcome measurements are shown for the intervention (*n* = 20) and control (*n* = 20) groups in Table 1, showing that the sample was homogeneous. In addition, the sex distribution between both groups did not present statistically significant differences (*p* = 0.748; χ^2^ = 0.417), showing 7 (35%) male and 13 (65%) female patients in the intervention group as well as 9 (45%) male and 11 (55%) female patients in the control group.

### 3.2. Between-Group Comparisons for Outcome Measurement Differences

The comparison for outcome measurement differences after treatment between the intervention and control groups is presented in Table 2, showing statistically significant differences (*p* < 0.05) with an effect size that varied from medium to large (*d* = 0.71–2.30) for greater thickness of the left and right hemi-diaphragms at T^ins^ and T^ins-exp^, as well as higher PI_max_ and decreased NRS, CSI and RMQ scores in the intervention group with respect to the control group. Nevertheless, the rest of the outcome measurements did not show any statistically significant differences (*p* > 0.05) between both intervention and control groups.

### 3.3. Multivariate Linear Regression Models

Multivariate linear regression models for the prediction of the outcome measurement differences which showed statistically significant differences between the intervention and control groups are presented in Table 3. After treatment, the increases in the thickness of the left and right hemi-diaphragms at T^ins^ and T^ins-exp^ and PI_max_, as well as the decrease in the NRS and RMQ scores, were only predicted by the proposed intervention (*R*^2^ = 0.118–0.552). In addition, the decrease in the CSI scores was only predicted by higher PPT at baseline (*R*^2^ = 0.266). Thus, the rest of the independent variables such as descriptive and baseline data were excluded (*p* > 0.05) from these prediction models due to these dependent variables not being influenced nor predicted by the descriptive data or other outcome measurements at baseline considering the pre-established F probability values (*P*_in_ = 0.05; *P*_out_ = 0.10).

## 4. Discussion

According to the study’s purpose, an 8-week program of HAG seemed to provide beneficial effects on inspiratory muscle strength, diaphragm thickness at inspiration, pain intensity and disability in patients with non-specific chronic LBP with respect to non-intervention. To the best of our knowledge, the present study findings support the effectiveness and prediction of HAG to improve inspiratory muscle strength in addition to an increase in diaphragm thickness during breathing, and to reduce pain intensity, central sensitization and disability in patients who suffer from non-specific chronic LBP. In accordance with Calvo-Lobo et al. [28], the association between a thinner diaphragm at inspiration during normal breathing and the presence of non-specific LBP was used to state the hypothesis about the main role of the diaphragm as a key mechanism during HAG in order to improve pain intensity, central sensitization and disability.

This study may be considered as the first research in the literature reporting a prediction and a cause–effect relationship between HAG and the strength and thickness of the diaphragm muscle in patients with chronic non-specific LBP. According to Caufriez [33] and Rebullido and Pinsach [34], HAG comprised breathing cycles with lateral-costal inspiration, and slow deep maximum exhalation and maintenance of breathing after rib cage expansion named diaphragmatic aspiration. We hypothesized that the start of this diaphragmatic aspiration could provide an eccentric contraction of the diaphragm against the pressure and opening of the rib cage to maintain its stable horizontal position from 10 to 20 s of apnea. This sustained and controlled eccentric contraction could explain the increased thickness and strength of the diaphragm during inspiration and the lack of thickness changes during expiration [43]. Nevertheless, the authors advise clinicians that HAG should be applied with caution as the effects of repeated eccentric contractions may predispose patients to deterioration of the diaphragm muscle function and structural damage according to animal model research [44].

In line with our findings, prior studies have recently shown that pain intensity and disability were improved after HAG in patients who suffered from chronic non-specific LBP [4,45]. In addition, our study supports the idea that HAG may predict a reduction in pain intensity and disability in chronic non-specific LBP patients. The Oswestry questionnaire score was used as an inclusion criterion according to a prior study evaluating the diaphragm fatigability in low back pain [31]. Nevertheless, the RMDQ was used as the preferred outcome measurement instrument as this validated and reliable tool used to measure LBP disability only presents 24 items, while the Oswestry questionnaire presents 60 items [41]. Despite the fact that there is a lack of prior studies assessing central sensitization under HAG intervention, central sensitization reduction under this treatment may be justified by respiratory mechanisms mediated by the central nervous system [46]. Nevertheless, our proposed intervention did not improve PPT nor kinesiophobia. The non-existence of PPT differences may be due to the fact that PPT was assessed on the spinous process, as a bone reference according to this measurement seemed to be more sensible over soft tissues than over bone references [47]. In spite of the fact that kinesiophobia was reduced in the HAG group and increased in the control group, these differences did not reach significance or the clinical important difference of 4 points according to the TSK-11, which could be due to the control group following their usual physical activity during the 8-week follow-up [48].

Future studies should evaluate the effectiveness of HAG in other conditions different from LBP which may improve the diaphragm function and consequently the clinical course of these pathologies, i.e., patients who suffer from COVID-19 [49].

### Limitations

Some limitations should be acknowledged. First, the main limitation of the present study was that the control group did not receive any treatment. Consequently, the authors recognize that efficacy may not be accurately detailed as these changes could be attributed to other factors different from the proposed intervention. Second, a placebo group was not considered, and thus future studies should implement a placebo intervention in order to improve the study strength. In addition, the comparison of HAG versus the intervention proposed by management guidelines for chronic non-specific LBP should be investigated [50]. Thirdly, HAG was performed for 8 weeks, and a wash-out period was not applied to evaluate the study findings after the intervention. Finally, the sample size calculation was generally based on the effect size due to the lack of prior studies assessing the diaphragm thickness during HAG in patients with chronic non-specific LBP. Loss to follow-up was not estimated in the sample size calculation as a low sample size could easily be achieved in this prevalent condition and controlled directly by phone to ensure involvement during the 8-week follow-up and the two outcome measurement moments. Future studies should use the diaphragm thickness differences after HAG to perform more adequate sample size calculations including large sample sizes and loss to follow-up estimations.

## 5. Conclusions

An 8-week HAG intervention seemed to show beneficial effects and predicted an increase in diaphragm thickness and strength during inspiration, as well as a reduction in pain intensity, central sensitization and disability, in patients suffering from chronic non-specific LBP with respect to non-intervention. Our findings could suggest the main role of the diaphragm muscle as a possible mechanism of hypopressive exercises to improve chronic non-specific LBP. Nevertheless, the short-term follow-up and the lack of comparisons versus placebo or other interventions are the main limitations that should be considered in future studies.

## Figures and Tables

**Table 1:** Baseline data for both groups.

Baseline Data	Intervention (*n* = 20) Mean ± SD (95% CI)	Control (*n* = 20) Mean ± SD (95% CI)
Age (years)	23.25 ± 4.52 (21.13–25.36)	23.90 ± 7.36 (20.45–27.34)
Weight (kg)	66.02 ± 11.11 (60.82–71.22)	66.40 ± 11.63 (60.95–71.84)
Height (cm)	168.25 ± 8.44 (164.29–172.20)	167.85 ± 7.25 (164.45–171.24)
Left diaphragm thickness at T^ins^ (cm)	1.35 ± 0.41 (1.15–1.55)	1.37 ± 0.43 (1.16–1.58)
Left diaphragm thickness at T^exp^ (cm)	1.05 ± 0.37 (0.87–1.22)	1.12 ± 0.37 (0.94–1.29)
Left diaphragm thickness at T^ins-exp^ (cm)	0.31 ± 0.15 (0.24–0.38)	0.25 ± 0.13 (0.19–0.31)
Right diaphragm thickness at T^ins^ (cm)	1.35 ± 0.36 (1.17–1.52)	1.58 ± 0.60 (0.90–3.10)
Right diaphragm thickness at T^exp^ (cm)	1.07 ± 0.29 (0.93–1.20)	1.21 ± 0.43 (1.00–1.41)
Right diaphragm thickness at T^ins-exp^ (cm)	0.27 ± 0.22 (0.17–0.38)	0.36 ± 0.31 (0.22–0.51)
PI_max_ (%)	98.75 ± 25.79 (86.67–110.82)	96.70 ± 16.99 (88.74–104.65)
PPT (kg/cm^2^)	4.44 ± 1.50 (3.73–5.14)	5.49 ± 2.17 (4.47–6.51)
NRS (score)	6.10 ± 1.55 (5.37–6.82)	5.70 ± 2.17 (4.68–6.71)
CSI (score)	27.35 ± 11.74 (21.85–32.84)	24.75 ± 11.17 (19.51–29.98)
TSK-11 (scores)	21.85 ± 5.33 (19.35–24.34)	21.40 ± 5.15 (18.98–23.81)
RMQ (scores)	3.30 ± 2.40 (2.17–4.42)	2.90 ± 1.68 (2.11–3.68)
IPAQ (METs/min/week)	2547.63 ± 2279.95 (1480.67–3614.78)	3196.02 ± 2713.71 (1925.96–4466.08)

Abbreviations: CI, confidence interval; CSI, Central Sensitization Inventory; IPAQ, International Physical Activity Questionnaire; MET, metabolic equivalent of task; NRS, Numerical Rating Scale; PI_max_, maximal inspiratory pressure; PPT, pressure pain threshold; RMQ, Roland–Morris Questionnaire; SD, standard deviation; T^ins^, inspiration time; T^exp^, expiration time; TSK-11, Tampa Kinesiophobia Scale-11 items. For all analyses, *p* > 0.05 (with a confidence interval of 95%) was considered.

**Table 2:** Comparison of outcome measurement differences after treatment between intervention and control groups.

Outcome Differences after Treatment	Intervention (*n* = 20) Mean ± SD (95% CI)	Control (*n* = 20) Mean ± SD (95% CI)	Mean Difference (95% CI)	Statistics	*p*-Value	Effect Size (Cohen *d*)
Left diaphragm thickness at T^ins^ (cm)	0.24 ± 0.21 (0.14–0.34)	0.04 ± 0.13 (−0.02–0.10)	0.20 (0.08–0.32)	*U* = 94.500	**0.004** †	*d* = 1.14
Left diaphragm thickness at T^exp^ (cm)	0.04 ± 0.26 (−0.07–0.16)	0.05 ± 0.14 (−0.01–0.12)	−0.01 (−0.14–0.12)	*U* = 200.500	1.000 †	*d* = 0.04
Left diaphragm thickness at T^ins-exp^ (cm)	0.20 ± 0.21 (0.09–0.30)	−0.01 ± 0.07 (−0.04–0.02)	0.21 (0.10–0.32)	*U* = 62.000	<**0.001** †	*d* = 1.34
Right diaphragm thickness at T^ins^ (cm)	0.33 ± 0.32 (0.17–0.48)	−0.08 ± 0.26 (−0.20–0.04)	0.41 (0.21–0.60)	*t* = 4.364	<**0.001** *	*d* = 1.40
Right diaphragm thickness at T^exp^ (cm)	0.08 ± 0.34 (−0.08–0.24)	0.00 ± 0.20 (−0.09–0.09)	0.08 (−0.10 –0.26)	*t* = 0.890	0.379 *	*d* = 0.28
Right diaphragm thickness at T^ins-exp^ (cm)	0.26 ± 0.20 (0.16–0.36)	−0.07 ± 0.22 (−0.18–0.03)	0.34 (0.20–0.48)	*t* = 4.929	<**0.001** *	*d* = 1.56
PI_max_ (%)	20.45 ± 11.83 (14.91–25.98)	−2.80 ± 8.00 (−6.54–0.94)	23.25 (16.75–29.74)	*t* = 7.278	<**0.001** *	*d* = 2.30
PPT (kPa)	0.69 ± 1.48 (0.00–1.39)	0.42 ± 1.21 (−0.14–0.99)	0.27 (−0.59 –1.13)	*t* = 0.631	0.631 *	*d* = 0.19
NRS (score)	−2.20 ± 1.73 (−3.01–−1.38)	0.05 ± 2.11 (−0.93–1.03)	−2.25 (−3.48–−1.01)	*t* = −3.679	**0.001** *	*d* = 1.16
CSI (score)	−5.80 ± 8.69 (−9.86–−1.73)	0.15 ± 5.82 (−2.57–2.87)	−5.95 (−10.68–1.21)	*t* = −2.543	**0.015** *	*d* = 0.80
TSK-11 (scores)	−1.45 ± 4.17 (−3.40–0.50)	0.55 ± 4.44 (−1.52–2.62)	−2.00 (−4.75–0.75)	*t* = −1.467	0.150 *	*d* = 0.46
RMQ (scores)	−1.30 ± 1.94 (−2.21–−0.38)	0.10 ± 1.97 (−0.82–1.02)	−1.40 (−2.65–−0.14)	*U* = 276.000	**0.040** †	*d* = 0.71
IPAQ (METs/min/week)	−29.63 ± 1321.60 (−648.16–588.90)	−356.42 ± 1834.94 (−1215.20–502.35)	326.79 (−696.84–1350.43)	*t* = 0.646	0.522 *	*d* = 0.20

Abbreviations: CI, confidence interval; CSI, Central Sensitization Inventory; NRS, Numerical Rating Scale; IPAQ, International Physical Activity Questionnaire; MET, metabolic equivalent of task; PI_max_, maximal inspiratory pressure; PPT, pressure pain threshold; RMQ, Roland–Morris Questionnaire; SD, standard deviation; T^ins^, inspiration time; T^exp^, expiration time; TSK-11, Tampa Kinesiophobia Scale-11 items. * Student′s *t*-test for independent samples was used. † Mann–Whitney *U* test was applied. For all analyses, *p* < 0.05 (with a confidence interval of 95%) was considered as statistically significant (bold).

**Table 3:** Multivariate linear regression models for the prediction of the outcome measurement differences which showed statistically significant differences between intervention and control groups.

Outcome Measurement Differences	Model (β)	*R*^2^ Change	Model *R*^2^
Left diaphragm thickness at T^ins^ (cm)	0.450 −0.205 * Group	0.250 ‡	0.250
Left diaphragm thickness at T^ins-exp^ (cm)	0.414 −0.214 * Group	0.312 ‡	0.312
Right diaphragm thickness at T^ins^ (cm)	0.740 −0.410 * Group	0.334 ‡	0.334
Right diaphragm thickness at T^ins-exp^ (cm)	0.217 −0.341 * Group	0.390 ‡	0.390
PI_max_ (%)	43.700 −23.250 * Group	0.582 ‡	0.582
NRS (score)	−4.450 +2.250 * Group	0.263 ‡	0.263
CSI (score)	−13.340 +2.117 * PPT at baseline	0.266 ‡	0.266
RMQ (score)	−2.700 +1.400 * Group	0.118 †	0.118

Abbreviations: CSI, Central Sensitization Inventory; NRS, Numerical Rating Scale; PI_max_, maximal inspiratory pressure; RMQ, Roland–Morris Questionnaire; PPT, pressure pain threshold; T^ins^, inspiration time; T^exp^, expiration time. * Multiply: Group (Intervention = 1; Control = 2). † *p*-value < 0.05 with a 95% confidence interval was considered. ‡ *p*-value ≤ 0.001 with a 95% confidence interval was considered.

## Data Availability

Data are available on request due to restrictions, e.g., privacy or ethical restrictions.

## Supplementary Materials

The following are available online at https://www.mdpi.com/article/10.3390/jcm10214983/s1, Supplemental File S1: Hypopressive abdominal gymnastics (HAG) program.
