# Supplementary material for: The Main Role of Diaphragm Muscle as a Mechanism of Hypopressive Abdominal Gymnastics to Improve Non-Specific Chronic Low Back Pain: A Randomized Controlled Trial
Source: J Clin Med. 2021 Oct 27;10(21):4983. doi: 10.3390/jcm10214983 (PMC8584898; doi:10.3390/jcm10214983)
Supplement: Supplementary file 1 [file jcm-10-04983-s001.zip › jcm-1397641-supplementary.pdf]

SUPPLEMENTAL FILE S1

HYPOPRESSIVE ABDOMINAL GYMNASTICS (HAG) PROGRAM	
WEEKS	EXERCISES
Week 1	Standing posture and Tailor Sitting posture
Week 2	Semi-Sitting posture and Tailor Sitting posture
Week 3	Supine position and Tailor Sitting posture
Week 4	Kneeling posture, Cuadruped posture and Genupectoral Sitting posture
Week 5	Standing posture and Tailor Sitting posture
Week 6	Kneeling posture and Cuadruped posture
Week 7	Semi-Sitting posture and Supine position
Week 8	Tailor Sitting posture and Genupectoral Sitting posture

HYPOPRESSIVE ABDOMINAL GYMNASTICS (HAG) EXERCISES

Standing posture

The participant stood with a neutral pelvis, his/her knees in flexion and his ankles in dorsiflexion. The feet were separated to the width of the pelvis. The upper limbs were placed with the shoulders in internal rotation, the elbows flexed to 90º and the hands were supported virtually on the iliac crests, with the wrists and fingers extended. The cervical spine was stretched, and the chin was posteriorized allowing full self-elongation. From this position, the body axis was advanced ventrally.

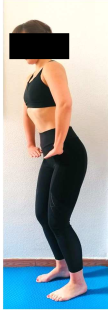

Standing posture.

Semi-Sitting posture

The participant was placed in a semi-sitting position with a neutral pelvis and sitting bones. The knees were slightly flexed and the feet were dorsiflexed. The lower limbs were aligned to the width of the hips. The upper limbs were positioned so that the shoulders were in an internal rotation, the elbows flexed at 90º. The hands in the finger and wrist extension position were virtually supported on the iliac ridges. A self-elongation and a double chin were performed.

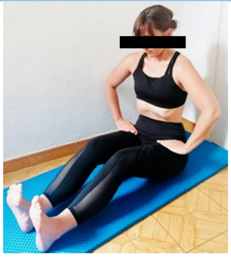

Semi-sitting posture

Tailor Sitting posture	
The participant was seated with a neutral pelvis. The lower limbs were crossed with the hips and knees bent. The upper limbs were positioned in the same way as in the previous exercises. A self-elongation and a double chin were performed.	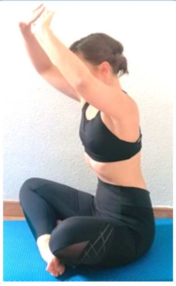 *Tailor sitting posture*
Kneeling posture	
The participant was placed on the knees that were bent with a dorsiflexion of the ankles with a neutral pelvis. The upper limbs were positioned in the same way as in the previous exercises. Self-elongation and a double chin were performed.	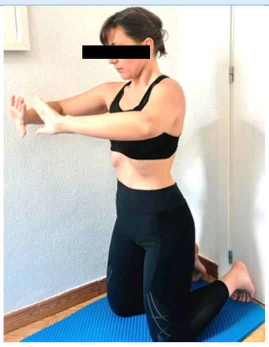 *Kneeling posture*
Supine position	
The participant was positioned lying face up with a neutral pelvis and the right leg crossed over the left. The knees were semi-flexed with the ankles dorsiflexed. The shoulders were in internal rotation with the elbows and wrists extended. The right hand was placed under the left one. A self-lengthening and a double chin were performed.	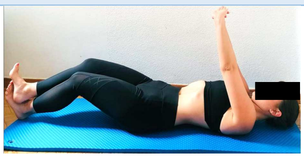 *Supine position.*
Cuadruped posture	
The participant was positioned in a quadruped position with a neutral pelvis, leaning on the knees and hands. The hip, knees and ankles were bent. The shoulders were in internal rotation with elbows slightly bent and wrists and fingers extended. Scapula abduction, self-elongation and double chin were performed.	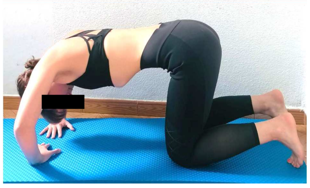 *Cuadruped posture*
Genupectoral Sitting posture	
The participant was positioned in the genupectoral sitting position with neutral pelvis. The buttocks were supported on the heels. In this position, the forehead, elbows and the front of the forearms were supported on the ground. Self-elongation was performed by pulling the pelvis back.	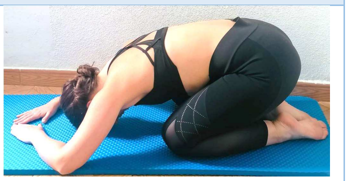 *Genupectoral sitting posture*
